# A novel aptamer-based dNTP assay reveals that intact HIV virions are highly stable and do not contain enough dNTPs to support DNA synthesis

**DOI:** 10.1128/jvi.00564-25

**Published:** 2025-07-15

**Authors:** Urja Biswas, Cynthia Bernal, Ruofan Wang, Jeffrey J. DeStefano

**Affiliations:** 1 Cell Biology and Molecular Genetics, Bioscience Research Building, University of Maryland 1068 , College Park, Maryland, USA; Icahn School of Medicine at Mount Sinai, New York, New York, USA

**Keywords:** virion structure, dNTP assay, reverse transcription, HIV, IP6

## Abstract

HIV virions contain high concentrations of IP6 (inositol hexakisphosphate), which stabilize capsids by binding to pores that allow dNTPs to import into capsids during infection. The concentration of dNTPs in virions, however, is not known. The low cellular concentrations (~5 µM each) predict only ~1 copy of each dNTP in a virion, unless a mechanism for concentrating exists, as it does for IP6. Reports indicating significant DNA synthesis occurring in virions would require larger amounts of packaged dNTPs or import of dNTPs. We developed a novel aptamer-based assay capable of detecting 1 femtomole of dNTP, enabling dNTP measurements in virions. Virions from HEK 293T cells contained dNTPs with dTTP >dCTP ≈ dATP >dGTP. This order was also observed in uninfected cells. Concentrations of dNTPs in virions were 2-fold to 4-fold lower than in cells, although it was unclear if this resulted from uncertainties associated with estimating virion and cell volumes or real differences. Virions were highly stable and did not “leak” dNTPs as associated dNTPs were not released, even after incubation at 60°C for 90 min or several freeze-thaw cycles, a result that implies diffusion-mediated import of dNTPs into virions is also unlikely. Overall, results show that dNTPs in intact virions are at or below concentrations in cells, and even the most abundant dNTP (dTTP) is present at ~1 copy per virion. This precludes significant DNA synthesis in intact virions, although it is important to note that our assays may not represent all virion states. The results support a mechanism where dNTPs are passively captured during virion budding.

**IMPORTANCE:**

HIV virions presumably package small molecules (e.g., amino acids, cations, and nucleotides) passively during budding. Therefore, they would be present in virions at cellular levels, unless there is a mechanism for concentration or exclusion of the molecule (e.g., inositol hexakisphosphate (IP6), which stabilizes the viral capsid, is present at several-fold greater concentration in the virion compared with cells). Reports of DNA synthesis occurring in HIV virions suggest that HIV may also package dNTPs at levels much higher than predicted by their cellular concentrations, or virions are permeable to dNTPs. However, we show here that virion dNTP levels are at or below cellular levels with 1 or fewer molecules of each dNTP in an average virion. Furthermore, virions did not leak dNTPs when exposed to elevated temperatures or freeze-thawing, suggesting that they were not permeable. The results suggest that virions demonstrating significant DNA synthesis may be structurally altered relative to those measured here.

## INTRODUCTION

Several studies have examined the protein and nucleic acid content of purified HIV virions, revealing that, in addition to viral proteins, virions typically incorporate several host-derived proteins and may also contain host RNA (1–4). However, analysis of the small molecule content in virions, such as nucleotides and divalent cations, remains largely unexplored. This is due, at least in part, to the difficulty in detecting and measuring these constituents. The small size of viruses (typically less than one millionth the volume of a cell) makes it difficult to recover sufficient material for measurements. This is further complicated by complexities associated with obtaining “pure” virus particles, as many viruses are similar in size and density to microvesicles and exosomes that are secreted by cells (5–7).

Immunoaffinity or protease treatment approaches combined with ultracentrifugation (7) and iodixanol gradients have all been successfully used to separate HIV from exosomes (5, 8, 9). These methods yield enriched virus particles that retain at least some infectivity (excluding protease treatment) (5, 10). Although these approaches have been used to examine the protein content of HIV particles (2), it is not clear to what extent specific small molecules are retained by the particles, as these methods often involve extensive manipulation and prolonged incubation times.

One small molecule that has been quantified in HIV virions is IP6 (inositol hexakisphosphate). This cellular molecule is required for viral capsid formation and stabilizes the mature HIV capsid (11, 12). The negatively charged IP6 molecule binds to the mature virus capsid with the hexameric assembly of Gag contributing 6 arginines that form a ring/pore with IP6 occupying the center of each ring and stabilizing the pore (13, 14). Each virion contains approximately 300 IP6 molecules, closely matching the ~250 hexamer sites on the HIV capsid (12). This work showed that IP6 is highly concentrated in virions relative to its cellular concentration. The role and potential mechanism of IP6-stabilized pores in dNTP import have been confirmed and remain an active area of research (15, 16).

The high concentration of IP6 in virions, along with its tight binding to the capsid, made it easier to quantify its levels. In contrast, dNTPs are present at low µM concentrations in dividing cells (17, 18), and unless they are also concentrated in virions, quantification would be more challenging. At approximately 1–5 µM (depending on dNTP and cell type), and with an HIV virion volume of ~5.2 × 10^−19^ liters (12, 19), there would be on average about 0.3–1.6 copies of each dNTP in a virion. There have been reports of significant viral DNA synthesis products occurring in HIV virions (20–24), and some have concluded this can result from active reverse transcription within virions (25–27), as opposed to reverse transcription in producer cells. The latter is known to occur with hepadnaviruses and spumaviruses (also retroviruses) (28, 29). Others, based on experiments showing that specific viral mutations dramatically increase virion DNA content, have suggested that DNA observed in HIV virions most likely results from aberrant processes occurring in producer cells, and the resulting viruses are defective (30). Furthermore, based on cellular concentrations, there should not be enough dNTPs in a virion to allow significant DNA synthesis (30). One possibility to explain DNA synthesis occurring in intact virions would be that they are permeable to dNTPs, and reports have indicated that the C terminus of the HIV-1 transmembrane protein (gp41) may be involved in permeabilizing the virion and allowing nucleotide uptake (31, 32). Attempts to measure dNTPs in virions have been rare. One report with Rous Sarcoma Virus (RSV), a retrovirus of similar size to HIV, indicated that RSV virions contain various enzymes and 50 to >100 copies of specific dNTPs (33). It should be noted that these experiments were performed before the concentrations of nucleotides in cells had been accurately measured and before exosomes were discovered. These results suggest that dNTPs are at much higher concentrations than would be expected in virions. However, although the mechanism for IP6’s enhanced concentration in the virion is clear, there is no known mechanism to account for increasing the concentration of dNTPs in virions. Furthermore, rATP, which is the most abundant nucleotide in cells and for which highly sensitive quantification assays are available, does not appear to be more concentrated in virions (12). In addition, it competes equivalently with dATP and other nucleotides for association with the capsid, suggesting that deoxynucleotides do not have a competitive advantage or disadvantage for accessing the capsid (12). Based on the available data, we hypothesized that dNTPs would be incorporated into virions at concentrations similar to their cellular concentrations and would therefore be at levels too low to support significant DNA synthesis.

To address these questions further, we measured the level of dNTPs in HIV virions, taking advantage of a highly sensitive novel aptamer-based dNTP assay developed in our lab. This assay can quantitatively detect dNTPs at concentrations as low as 0.05 nM (1 femtomole in a 20 µL) in solution. Our results showed that dNTP concentrations in HIV virions derived from the transfection of HEK 293T cells were lower than those measured in cells, based on estimated cell and virus volumes. However, it is important to note that cell and virus volume estimates exhibit high variance due in part to their pleiomorphic nature (12, 19, 34–40). Like cellular dNTP pools, dTTP was at the highest concentration in virions, whereas dATP and dCTP were at similar levels, and dGTP was barely detectable above background levels. Isolated virions were highly stable, demonstrating no leakage of dNTPs during freeze-thawing and requiring incubation at high temperatures (>60°C) to release dNTPs. Overall, the results demonstrate that dNTPs in virions are present at near cellular or lower levels, and the virion forms a stable leak-proof container.

## RESULTS

**Fig 1:**
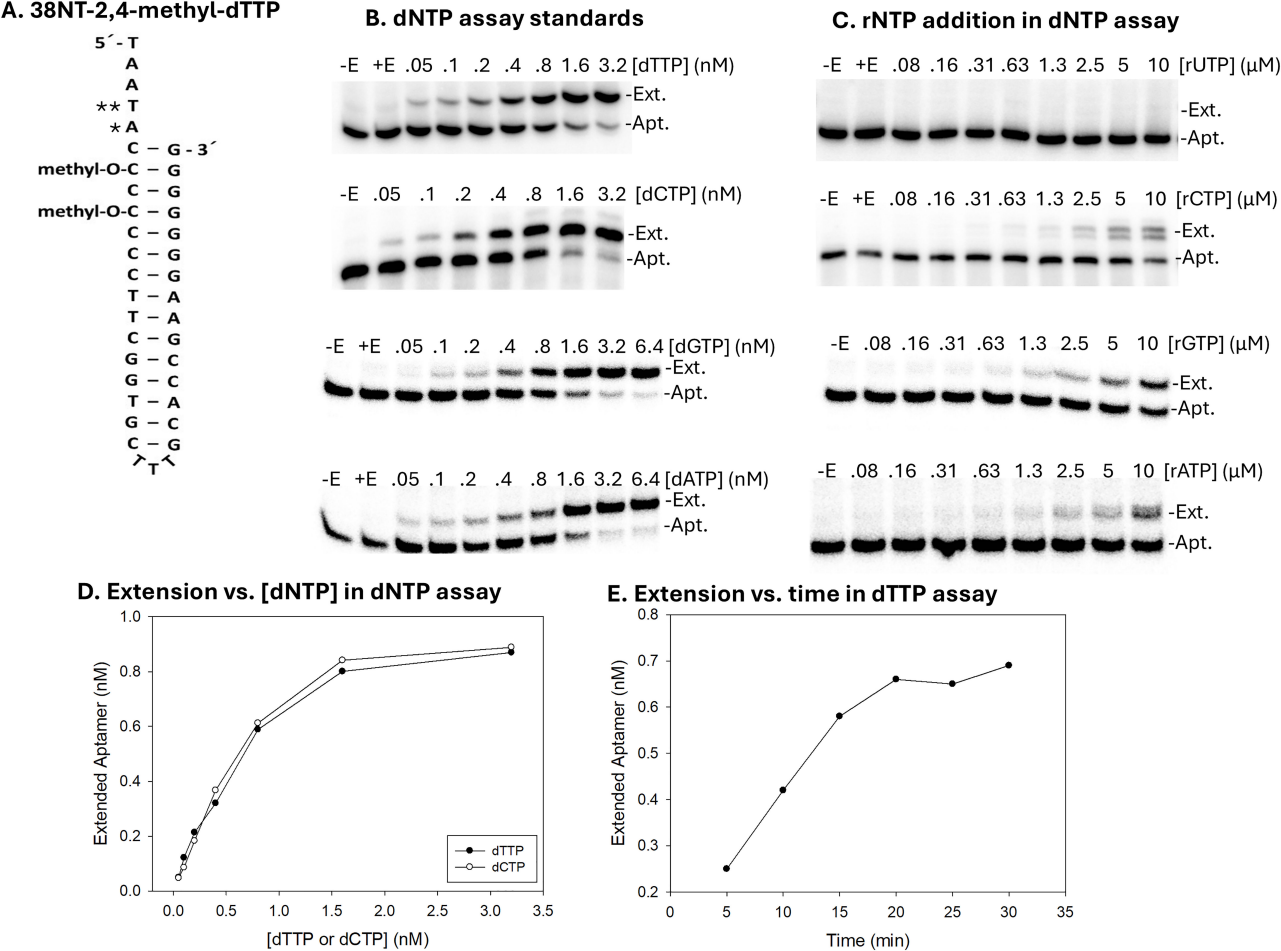
(A–E) Evaluation of aptamer dNTP assays. (A) The 38-nt aptamer used to quantify dTTP is shown. Aptamers were 5´-end-labeled with P-32 and present at 1 nM in assays. Four aptamers were used in assays to measure each dNTP. They are designated 38NT2,4-methyl-dNTP with the “dNTP” indicating which dNTP they measure. 38NT2,4-methyl-dTTP is shown. For dCTP, dATP, and dGTP versions, the “*” and “**” marked bases were as follows: dCTP: *dGTP, **dTTP; dATP: *dTTP, **dATP; and dGTP: *dCTP, **dTTP. (B) Standard assay for measuring all 4 dNTPs is shown. Extended (Ext.) and unextended aptamer (Apt.) positions are indicated. Marked above each lane is the amount of dNTP added to the reaction. Lanes with no dNTP added are shown in the absence (-E) and presence (+E) of the HIV RT enzyme used to extend the aptamer. Refer to Materials and Methods for details on reaction conditions. (C) dNTP assays were performed as in panel B except dNTP was replaced by the matching rNTP at the concentrations indicated. (D) Sample standard curves generated for dTTP and dCTP from the experiments shown in panel B. See Materials and Methods for an explanation of how the amount of extended aptamer was determined. (E) Time course of extension with the 38NT2,4-methyl-dTTP using 0.8 nM dTTP.

Based on cellular estimates of dNTP concentrations and the estimated HIV virion volume, ~1 molecule of each dNTP would be expected in a virion without a mechanism to concentrate or exclude them (see Introduction). Assuming one molecule in a virion, this would lead to a concentration of 0.083 nM for a solution of 20 µL with 10^9^ virions (1.66 femtomoles in 20 µL). This level is below the sensitivity limits of current dNTP assays, which would have required large amounts of HIV virions to produce accurate results (41–48). To overcome this limitation, we modified a previous radioactivity-based assay that uses HIV RT to incorporate nucleotides on a primer template (17, 49). This approach was coupled with a primer-template mimicking aptamer construct selected and designed in our lab for high-affinity binding to HIV RT (50). This construct, termed 38NT2,4-methyl, has been used in several publications to investigate the structure of HIV RT in complex with primer-template and various drugs (51–54). An important advantage of the aptamer over other primer-template constructs is that it does not require chemical or antibody cross-linking for crystallization and Cryo-EM analysis. The aptamer binds to HIV RT with pM affinity, which is approximately 2 orders of magnitude more tightly than binding to typical primer templates. To enable dNTP quantification, the template overhang of 38NT2,4-methyl was modified to produce 4 versions of the aptamer that would direct the incorporation of different dNTPs at the first position after the 3' terminus (Fig. 1A). The second template overhang nucleotide was also changed in some constructs to allow the incorporation of a second chain-terminating nucleotide. Extension of each version of the aptamer produced a product two nucleotides longer than the starting material that could be separated on a denaturing PAGE gel. With this system, using 1 nM final concentration of 5' end P-32 radiolabeled aptamer, a detection limit of ~1 fmol of dNTP and a dynamic range from 0.05 to ~1 nM dNTP concentration was achieved (Fig. 1B). Note that the range is small, but this was necessary to achieve the high sensitivity needed for virion measurements. The assay can also be used with higher amounts of aptamer (we investigated 5 and 10 nM levels), which improves the range while decreasing the sensitivity (data not shown). Finally, a version of the assay using fluorescently labeled aptamer was also tested (Fig. S1). This approach has the advantage of not requiring radioactivity. Since fluorescence emissions yield a tight band on gels, resolution was high with this approach. The main drawback was a ~ 40-fold loss of sensitivity, reducing the detection limit to about 40 femtomoles, which is on par with many other assays (41–48). One advantage was that the dynamic range increased by more than an order of magnitude compared with the radioactivity-based assay.

Each aptamer (designated 38NT2,4-methyl-dNTP, with dNTP being either dTTP, dCTP, dATP, or dGTP) was assessed by titration with standard concentrations of the nucleotide it measures and 50 nM or 500 nM of a chain-terminating nucleotide. Either dideoxy nucleotides (50 nM) or acyclonucleotides (500 nM) were employed as terminators, with the former used for 38NT2,4-methyl-dTTP and 38NT2,4-methyl-dATP and the latter used for the dCTP and dGTP aptamer versions. The reason for using the different terminators was based on better separation on denaturing PAGE gels. In particular, the dCTP and dGTP versions showed less separation between extended and non-extended aptamer on the gels. Controls that included the terminator in the absence of the added nucleotide being measured showed that terminators were almost exclusively incorporated in the second position after incorporation of the correct nucleotide at the first position and therefore did not interfere with quantification. Fig. 1B shows a typical set of experiments used to measure the concentration of dNTPs and evaluate the aptamer assays. Experiments shown are with 38NT2,4-methyl-dTTP in the top panel and dCTP, dGTP, and dATP assays below. There was a nearly linear response to the concentration of added dTTP or dCTP (similarly for dGTP and dATP) from 0.05 to about 0.6 nM followed by the curve leveling off as the concentration of added dNTP exceeded the amount of aptamer (1 nM) (Fig. 1D). Up to about 0.6 nM, the amount of aptamer extended was approximately equal to the amount of dNTP added to the assay for both dTTP and dCTP, whereas dATP and dGTP assays showed modestly lower extension. This indicated that most of the dNTP substrate was incorporated. This is similar to what was observed in the assay from which the aptamer assay was derived (17, 49). As these assays appear to work by complete or nearly complete depletion of the dNTP substrate, employing a polymerase without 3′−5′ proofreading exonuclease activity and devoid of exonuclease contamination is pivotal. Maximum extension was observed after about a 20 min incubation, and all assays in this report were performed for 25 min (Fig. 1E). HIV RT can add ribonucleotides in place of dNTPs during DNA synthesis (55, 56). The level of selectivity for dNTPs in a previous report varied depending on the particular nucleotide, with selectivity against rUTP and rCTP being >10,000-fold, rGTP about 600-fold, and rATP only 20-fold (56). Results with our assay also showed high discrimination against rUTP. An assay in which dTTP was replaced by rUTP showed no incorporation of rUTP, even when added at 10 µM (Fig. 1C, top panel). Interestingly, for rCTP, rGTP, and rATP, ~1 µM of each with the matched aptamer resulted in a level of extension equivalent to about 0.05–0.1 nM of the corresponding dNTP (Fig. 1C lower panels). Therefore, the current assay demonstrates high discrimination against rUTP, and about equivalent, and somewhat lower discrimination against other rNTPs.

The above results suggested that although the level of rNTPs may be much higher in the virion as they are in cells (100-fold or higher compared with dNTPs in cells [55, 57]), they would not reach levels that would significantly interfere with dNTP quantification in the assays. To test this further, assays with a standardized level of the dNTP being measured or with no added dNTP were conducted in the presence of all 4 rNTPs and 3 dNTPs (excluding the dNTP being measured) that were adjusted to match cellular levels based on our rATP and dNTP measurements in cells (see description in the supplemental material). The results from these assays showed that there was little or no interference with the quantification of dTTP and dGTP even when other rNTPs and dNTPs were added at 16-fold above their relative concentrations in cells (Fig. S2). In contrast, assays for dATP and dCTP were modestly affected by the presence of excess rNTPs and dNTPs, but only when they were added at 4-fold to 16-fold above expected cellular concentrations. The results indicate that the presence of other nucleotides in the dNTP assays does not interfere with quantification within the range expected to be present in the assays.

### Production of virions for dNTP analysis

Having developed an assay capable of quantifying minute levels of dNTPs, we focused on isolating intact virions with minimal cellular dNTP contamination. HEK 293T cells transfected with viral vectors are perhaps the most common approach for producing HIV. Despite not being a T cell-derived line, HEK 293T cells produce large quantities of viruses that are highly infectious and outperform T cell lines for yield and integrity of the virions (58). Furthermore, HEK 293T cells have been used for many experiments (for example, this line was used to quantify IP6 levels in HIV [12]) and are well-characterized for nucleotide content among other things (18, 59). After growth in exosome-depleted serum, followed by filtration, a single sucrose cushion step, and subsequent concentration, viruses were easily isolated in a few hours without any freezing steps. Mock-transfected cell controls processed the same way showed very low levels of contaminating dNTPs in assays (see below), indicating that the levels of contaminating exosomes or microvesicles were low. Embryonic cell lines generally produce fewer exosomes than lymphoid cell lines; hence, this was not surprising (7). We were able to detect low levels of dNTPs in controls in assays where larger amounts of control material were added (Fig. S3), indicating that there are likely some exosomes/microsomes in the preparations, but not enough to significantly affect virus readings.

### Measurement of dNTP levels in HIV virions

**Fig 2:**
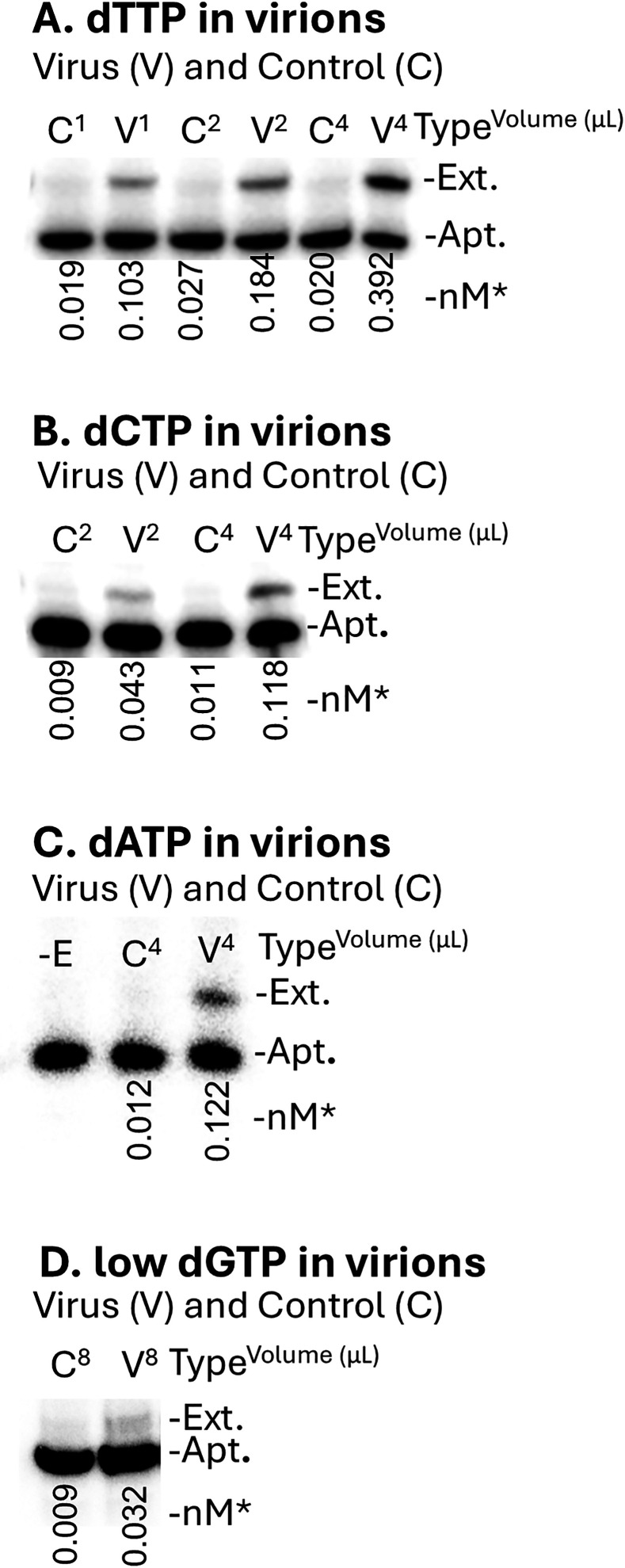
(A–D) Isolated virions contain detectable amounts of dNTPs. Extension of aptamers 38NT2,4-methyl-dTTP (A), 38NT2,4-methyl-dCTP (B), 38NT2,4-methyl-dATP (C), and 38NT2,4-methyl-dGTP (D) with isolated virion samples (V) or mock-transfected control (C) samples. The amount of virus or matched control samples is indicated by the superscript in microliters. In this set of assays, 1 µL corresponded to about 2.5 × 10^9^ virions. For the dGTP assay (D), 8 µL of V or C sample was added. *Numbers under lanes indicate the amount of extended aptamer in “nM” out of a total of 1 nM added to each reaction and determined as described in Materials and Methods. Other markings are as described in Fig. 1.

The level of various dNTPs in HIV was measured after purifying virions through a sucrose cushion as described in Materials and Methods. Virions were heated to 95°C for 2 min in buffer (20 mM HEPES pH 7.4, 150 mM NaCl) containing 0.1% Tween 20. Subsequent analysis showed that Tween 20 was not required for dNTP release. The 38NT2,4-methyl-dNTP aptamers (Fig. 1A) were used to measure the levels of all 4 dNTPs in viral particles. The results with each aptamer in experiments with matching mock-infected controls and with different amounts of added virions (as indicated) are shown in Fig. 2. Virion levels were calculated from p24 ELISAs and an estimate of 10^4^ virus particles per picogram of p24. Although controls showed low levels of dTTP, levels in virion preparations were easily detected and increased with increasing amounts of sample (Fig. 2A). Using an estimated virus volume of 5.2 × 10^−19^ L per virion (12), an average value (mean ± S.D.) from several experiments for dTTP of 2.09 ± 0.88 µM was calculated (Table 1 summarizes the results for all dNTP analyses). Virions had a lower but still easily detectable level of dCTP (Fig. 2B) and dATP (Fig. 2C). In contrast, the level of dGTP was very low. Although dGTP levels were consistently above controls, they did not typically rise to the level of the lowest standard (0.05 nM), making it difficult to calculate dGTP amounts with any certainty. An assay with dGTP is shown in Fig. 2D. Note that there was twice as much virus and mock-transfected control material added in the dGTP assay compared with the highest amounts added for other nucleotides in Fig. 2 and still just trace amounts of dGTP were detected over the control.

**TABLE 1:** Statistics for cell and virion dNTPs experiments

Nucleotide	293T cell concn (μM)^*a*^	Virion concn (μM)^*b*^	Ratio cell/virion
rATP	891 ± 426	183 ± 107	4.9
dATP	1.02 ± 0.27	0.58 ± 0.19	1.8
dGTP	0.41 ± 0.14	TLTD^*c*^	ND^*d*^
dCTP	1.65 ± 0.41	0.45 ± 0.22	3.7
dTTP	4.67 ± 1.68	2.09 ± 0.88	2.2

^*a*^ Based on 293T cell volume of 1.77 × 10^−12^ L/cell (12); avg ± S.D. from at least three independent experiments.

^*b*^ Based on HIV-1 virion volume of 5.2 × 10^−19^ L/virion (12); avg ± S.D. from at least three independent experiments.

^*c*^ TLTD, too low to determine. dGTP was detected in virions (see Fig. 1), but the concentration was too low to accurately determine.

^*d*^ ND, not determined.

Finally, a commercially available assay was used to calculate the concentration of rATP in virions and indicated that it was at a much higher concentration than dNTPs at 183 ± 107 µM (Table 1). This is expected, given rATP’s abundance in cells (55, 57). Overall, these results show that virus particles on average contain ~1 copy of dTTP and less than 1 copy of the other 3 dNTPs.

### Measurement of dNTP levels in HEK 293T cells

**Fig 3:**
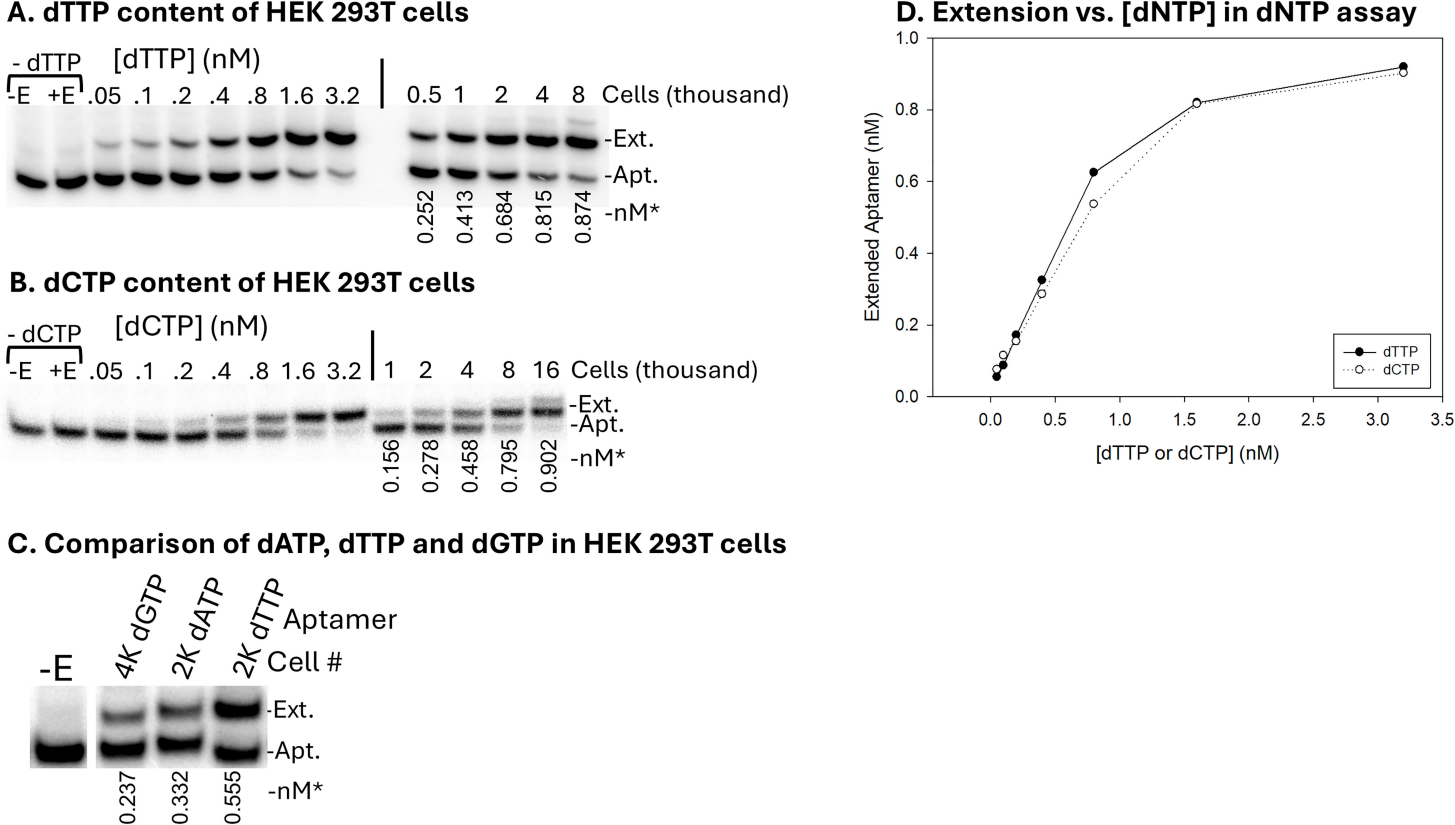
(A–D) Aptamer dNTP assay results from HEK 293T cells. (A) The 38NT2,4-methyl-dTTP aptamer used to quantify dTTP is shown in an assay with standard amounts of dTTP on the left. This is the same panel that was used in Fig. 1B under “dNTP assay standards” for dTTP. See Fig. 1 for more details. On the right, the dTTP assay is shown with different amounts of material (cell number as indicated) isolated from HEK 293T cells. See Materials and Methods for further details. (B) The 38NT2,4-methyl-dCTP aptamer used to quantify dCTP is shown in an assay with standard amounts of dCTP on the left. On the right, the dCTP assay is shown with different amounts of material (cell number as indicated) isolated from HEK 293T cells. (C) A comparison of dGTP, dATP, and dTTP in cells is shown. Note that 2,000 cells were used for dATP and dTTP and 4,000 cells for dGTP. The “-E” lane was with the dTTP aptamer and comes from another location on the same gel. (D) Sample standard curves generated for dTTP and dCTP from the experiments shown in panels A and B. *Numbers under lanes indicate the amount of extended aptamer in “nM” out of a total of 1 nM added to each reaction and determined as described in Materials and Methods. Refer to Fig. 1 for a description of other labels.

The level of the various dNTPs in HEK 293T cells was measured in mock-transfected cells removed from 100 mm plates by trypsinization (see Materials and Methods). Pelleted cells were counted with a hemocytometer, extracted with methanol, and assayed with the aptamer constructs. For some constructs, a small amount of extension beyond the terminator position occurred (Fig. 3A and B, highest cell amounts). Adding more terminators typically prevented this, but we chose to use the same amounts used for the virus assays for consistency. Any material extended beyond the terminator position was considered “extended” with the correct nucleotide in calculations. Like virus preparations, the levels of dTTP (Fig. 3A), dCTP (Fig. 3B), and dATP (Fig. 3C) in cells were the highest, whereas dGTP (Fig. 3C) was lower (Table 1). Concentrations in cells compared with viruses were approximately 1.8-fold, 2.2-fold, and 3.7-fold greater for dATP, dTTP, and dCTP, respectively. dGTP could not be compared, as it was not quantified in virion (see above). Overall, the calculated concentrations and relative levels of dNTPs in HEK 293T cells were close to the levels determined by others using a radiation-based assay and mock-transfected HEK 293T cells (18), whereas they were lower than levels determined in other radiation- and non-radiation-based approaches (43, 44). A comparison of our results with others for HEK 293T cells, lymphocytes, and T cells is shown in Table S1. It is important to note that the level of dNTPs in cells can vary depending on growth conditions and the growth state of the cells. Therefore, it is difficult to directly compare our results with others. The concentration of rATP was also measured and was just below 1 mM (Table 1). This level is on the low side of other determinations in cells which range from about ~0.5–10 mM (60, 61). Overall, cells had higher concentrations of dNTPs and rATP than viruses. However, as noted in the Introduction, cell and virus volumes show high variability, and estimating the number of intact HIV virions from p24 levels is complicated by imprecisions in p24 assays. The state of the virus particles (i.e., intact vs damaged) is also unknown, although the experiments below indicate that isolated virions are highly stable. Despite uncertainties in this analysis that are inherent and largely unavoidable, it was clear from the results that viruses do not have concentrations of dNTPs that are significantly higher than those in cells.

### Virus particles retain infectivity during processing with manipulations leading to an approximately 2-fold to 3-fold decrease in relative infectivity

Virus particles from the supernatant of transfected cells were compared with the processed particles by titering on HeLa TZM-bl cells. The results showed that the virus from the supernatant was on average 2.6 ± 0.4 (3 exp. ± S.D.) times more infectious based on TCID_50_/p24 content analysis (Table S2). Overall, this was viewed as a modest loss of infectivity. It may have resulted from the loss of viral envelope proteins from the virion surface. Viruses derived from pNL43 show a relatively high tendency to lose envelope protein compared with primary isolates (62, 63). As our purification process was less intensive than that used by others to accurately measure IP6 levels in HEK 293T-derived HIV virions (12), it is unlikely that it would have resulted in nucleotide leakage.

### Isolated virus particles form heat-stable shells that only release dNTPs upon heating to high temperatures and do not leak after repeated freeze-thaw cycles

**Fig 4:**
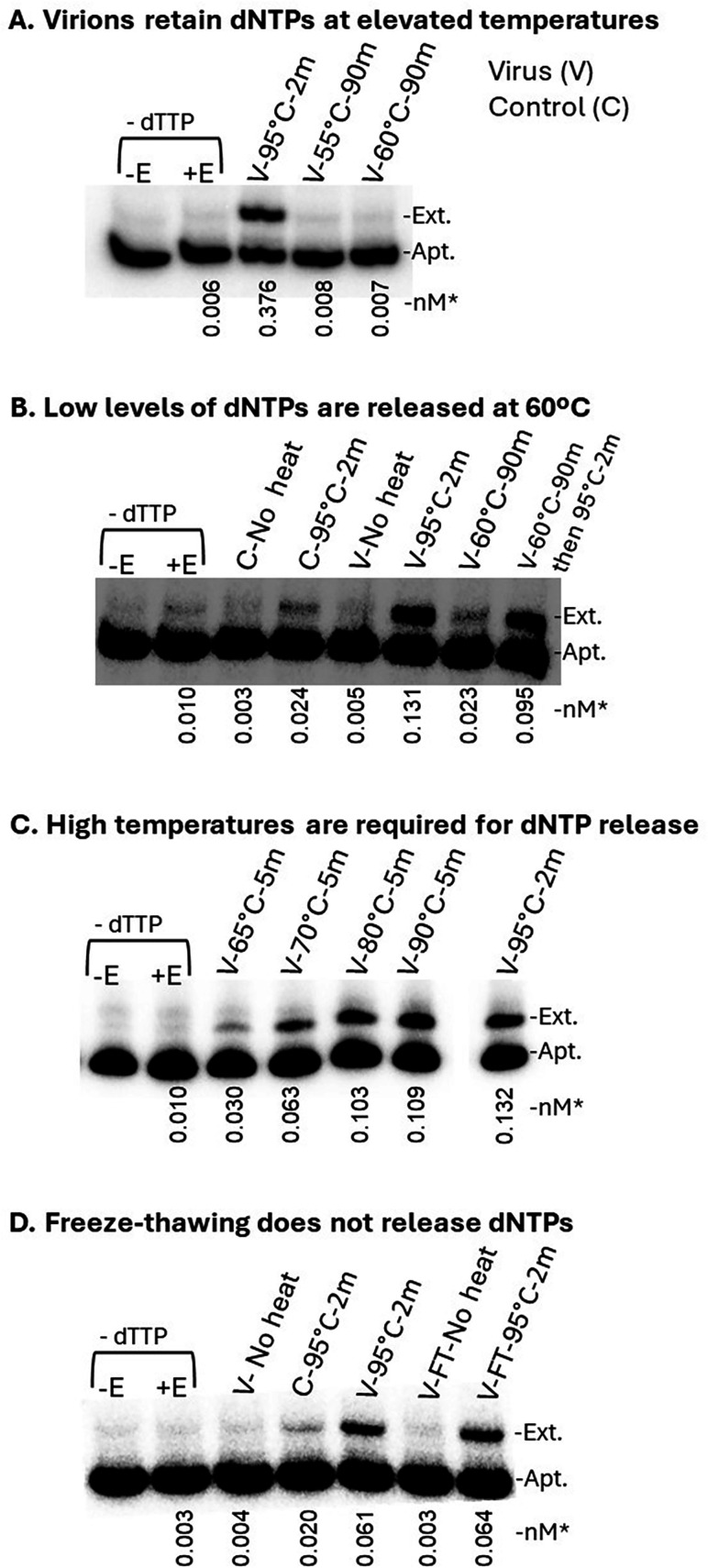
(A–D) Isolated HIV virions are heat-stable and freeze-thaw resistant. The 38NT2,4-methyl-dTTP aptamer (Fig. 1) was used to quantify dTTP in isolated virions that were incubated at different temperatures before assaying. The incubations were in the absence of detergent (see Materials and Methods). (A) Virions retain dNTPs during incubations at elevated temperatures. The standard preincubation, 95°C for 2 min, and two additional 90 min incubations at 55°C and 60°C are shown. (B) An overexposed gel reveals that heating virions at 60°C for 90 min releases a small amount of dNTPs and does not degrade dTTP in the virion. Labels are as above, except an additional sample with no preincubation (“no heat”) and a sample heated to 60°C for 90 min, then 95°C for 2 min is shown to confirm that dTTP was not degraded during the 90 min incubation. Mock-transfected control samples are also shown for comparison. (C) Temperatures above 60°C break virions and release dTTP. Virions were preincubated for 5 min at 65°C, 70°C, 80°C, and 90°C (as indicated), or for the standard 95°C for 2 min, then assayed for dTTP content as described in Materials and Methods. (D) Freeze-thawing virions does not cause the release of dNTPs. Isolated virions (V) or mock-transfected control samples (C) were heated or not heated (as indicated) prior to assaying for dTTP content. The two far right lanes were virions that were freeze-thawed (FT) in the absence of detergent (5 cycles of freezing at −80°C, followed by thawing at 37°C), then assayed for dTTP either without heating or after subsequent heating to 95°C for 2 min. *Numbers under lanes indicate the amount of extended aptamer in “nM” out of a total of 1 nM added to each reaction and determined as described in Materials and Methods. Refer to Fig. 1 for a description of other labels.

Assays for dNTPs were conducted by first heating the samples in 8 µL of buffer (20 mM HEPES, pH 7.4, 150 mM NaCl) for 2 min at 95°C (see Materials and Methods). This was necessary to release dNTPs from virions. In order to determine the stability of purified virions, the samples were incubated at several different temperatures for 90 min and then tested with 38NT2,4-methyl-dTTP to measure dTTP concentration. Tween-20 was omitted in these incubations, as it would have destabilized the particles. Incubating virions at 55°C or 60°C for 90 min did not cause the virions to release much dTTP (Fig. 4A). A second experiment to test this further is shown in Fig. 4B. Virion samples were incubated at 60°C for 90 min or at 60°C for 90 min and then 95°C for 2 min. The gel was intentionally over-exposed in an attempt to detect even very low levels of dTTP release. At this exposure level, a small amount of dTTP, approximately equivalent to what was observed in the mock-transfected control cell preparations heated to 95°C for 2 min, was observed after the 90 min incubation at 60°C. Virions incubated at 60°C for 90 min, then 95°C for 2 min showed high levels of dTTP approximately equivalent to the level observed with just a 2 min 95°C incubation of the virus sample. This indicated that the dTTP was not degraded during the 60°C incubation. Virions were then tested by incubating for 5 min at 65°C, 70°C, 80°C, or 90°C (Fig. 4C). Partial release occurred at 65°C and nearly complete release at about 80°C. These results suggest that despite virions being enveloped and containing a capsid held together by non-covalent bonds, they remain intact at surprisingly high temperatures.

Resistance to freeze-thawing was also tested. Purified virions were subjected to 5 cycles of freezing at −80°C, followed by thawing at 37°C. The level of dTTP in the virions was tested using the aptamer dTTP assay by direct assay of the freeze-thawed virions without heating to 95°C for 2 min, or after subsequent heating (Fig. 4D). The freeze-thawed, then heated virions had the same amount of dTTP as a control that was not freeze-thawed but was heated to 95°C for 2 min, whereas the freeze-thawed sample that was not subsequently heated was equivalent to the unheated virion sample, with no significant detectable dTTP. These results show that freeze-thawing did not release dTTP from the virions.

### Release of dNTPs from mock-transfected control samples indicated that they were derived from structures that are less heat stable than virions

**Fig 5:**
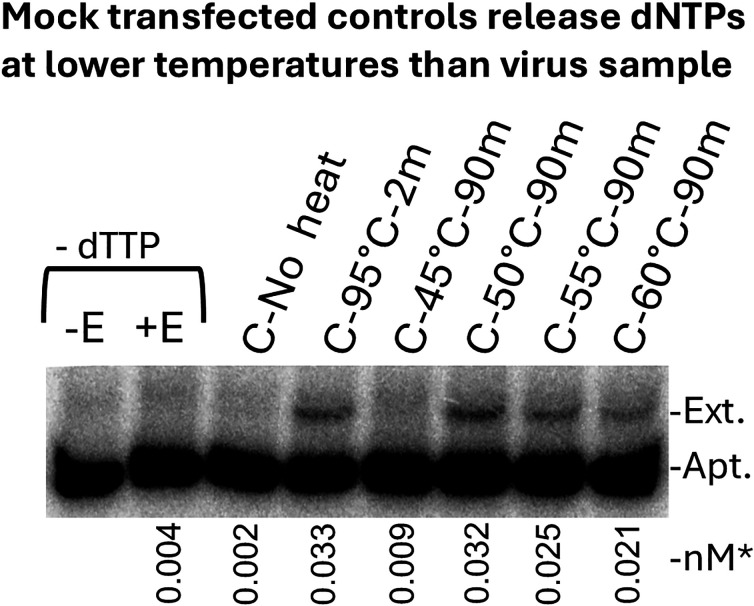
Release of dNTPs from mock-transfected control samples indicated that they are derived from structures that are less heat stable than virions. The 38NT2,4-methyl-dTTP aptamer (Fig. 1) was used to detect dTTP in mock-transfected controls that were incubated at different temperatures before assaying. The incubations were in the absence of detergent (see Materials and Methods), and the gel was overexposed to detect the low level of dTTP in the controls. Mock-transfected controls (C) were prepared as described in Materials and Methods. *Numbers under lanes indicate the amount of extended aptamer in “nM” out of a total of 1 nM added to each reaction and determined as described in Materials and Methods. Refer to Fig. 1 and 4 for a description of other labels.

In Fig. 4B above, overexposure of the gel revealed a clear release of dTTP in mock-transfected controls after heating to 95°C for 2 min. We note that the level of dTTP in controls was variable, and not all preparations contained detectable levels, but many did (see Fig. S3). We took advantage of this to investigate the nature of the structures that contained the dNTPs in the controls. Given the purification procedure, exosomes or microvesicles would be a likely source of these dNTPs, although no attempt was made to confirm this. To examine the effect of incubating samples at elevated temperatures, control samples were either not preincubated, heated to 95°C for 2 min to release all dNTPs, or preincubated at 45, 50, 55, and 60°C for 90 min, similar to the above experiments with virus samples. The 50, 55, and 60°C preincubations released about the same level of dTTP as the 95°C for 2 min sample (Fig. 5). This indicated that dNTPs in the controls are sequestered in structures that are less heat-stable than virions, as virions did not release significant amounts of dNTPs even at 60°C (Fig. 4A and B).

## DISCUSSION

In this report, we developed a novel, sensitive dNTP assay and showed that intact HIV virions contain low levels of dNTPs. Unlike IP6 (12), dNTPs are not concentrated in virions. The results suggest that most virions have perhaps a single molecule of dTTP and may also have a molecule of dCTP or dATP while rarely containing a molecule of dGTP. This would preclude any significant DNA synthesis in intact HIV virions. Virions were also highly stable, resisting freeze-thawing and releasing dNTP only upon heating to over 60°C. Although this does not necessarily mean they cannot take up dNTPs, this also seems unlikely. Previous reports of DNA synthesis in virions (25–27) are inconsistent with our observations, although it is possible synthesis could occur in damaged or misformed virus particles that allow the uptake of dNTPs. Our findings also do not exclude the possibility of DNA synthesis occurring in virus-producing cells prior to budding and virion maturation, although this would likely be an aberrant process for HIV as opposed to some other retroviruses (28, 29). Finally, we cannot exclude, nor do we have any evidence for, the possibility that there are other subsets of HIV virions that behave differently than the “intact” virions we isolated. These may or may not be infectious, and although purifying with “intact” virions, they do not contain dNTPs after the purification process (see below).

Other experiments with RSV suggested that there were 100 or more copies of some dNTP molecules in virions (33). Since RSV is similar in size to HIV, this would imply that the virus may have a mechanism for concentrating dNTPs in the virion, or the samples being measured were contaminated with cell-derived components. The latter seems more likely, as these measurements were performed decades ago before the presence of exosomes or microvesicles was understood. Other possibilities, like the cells the virus was derived from, had exceptionally high dNTP concentrations, are also unlikely as a level of 100 nucleotides in a virion, the size of HIV would correspond to a concentration of over 300 µM in cells. This would be more akin to the reported concentration for some rNTPs (e.g., rUTP or rCTP) in cells but is ~100 times greater than expected dNTP concentrations (18, 55, 57).

As expected, the concentration of rATP in virions was much higher than dNTPs at 183 ± 107 µM. However, this was about 5-fold lower than the measured concentration in cells (Table 1). Concentrations of dNTPs in virions were also consistently lower than cellular concentrations (Table 1). The amount of rATP in HIV virions was also estimated during the calculation of IP6 levels by another group (12). In that report, rATP in virus particles (~1.5 mM) was much higher than the value measured by us, but cellular levels were also much higher (~8 mM). Those differences could be related to the different assay and processing protocols used in the reports. However, both reports showed several-fold higher cellular concentrations of rATP compared with virions. Both reports used HEK 293T cells, and the same cellular and virus volume estimates were used; hence, this cannot account for the findings. One important note is that the measured concentrations of rATP in cells correspond to actively growing HEK 293T cells that are not producing virus particles. In contrast, the viruses were derived from cells actively producing HIV, which could potentially alter the level of rATP, or even the dNTP level, although the alterations would likely be modest. Transfected HEK 293T cells can produce HIV for days (although division and cell growth can be affected by transfection reagents) (64). This suggests that they are unlikely to have dramatically lower dNTP or rATP levels.

There may be several explanations for the lower concentrations of nucleotides in virions. For example, it is possible that the volume estimates used in our experiments for cells and/or viruses do not match with high precision to the available solute space in particles and/or cells. Cell volume calculations for HEK 293T cells (and many other cell types) are variable and are dependent on the growing state of the cells and approximations of their shape (12, 34, 38, 39). Retroviral virions are typically pleiomorphic, and volume calculations are based on the diameter of particles and the approximation of spherical morphology (19, 35–37, 40). Calculation of virion numbers in our experiments was also based on p24 content, which introduces other variables associated with the accuracy of the p24 assay and the assumption of ~10,000 virions per picogram of p24. Combined, these variables make it nearly impossible to estimate dNTP concentrations with high precision. A second possibility is that a significant number of virion particles are damaged, allowing small molecules to leak out after budding, during centrifugation, or during processing. The data from IP6 calculations (12) argue against this; however, IP6 is tightly bound to capsids. Hence, it may not rapidly leak out even if the viral envelope were damaged, and those experiments used VSV-G pseudotyped virus, which may have different membrane properties. Exposure of “leaky” virions to cell growth media could deplete dNTPs and rATP, especially since we found that the cell growth media used here contained dNTP concentrations that were below the sensitivity threshold of our dNTP assays (data not shown). Our experiments do show that purified virions containing dNTPs are not leaky and are highly stable, resisting elevated temperatures and freeze-thawing (Fig. 4). However, this does not directly address the possibility that a portion of the virions were “abnormal” or damaged during processing and leaked their contents prior to or during processing. These abnormal/damaged virions may retain their basic shape and purify along with undamaged “intact” virions, thus contributing p24, but not dNTPs or rNTPs, and therefore resulting in a misleadingly lower nucleotide concentration in virions compared with cells. The possibility that the so-called abnormal virions are “different” rather than abnormal cannot be ruled out by our work. It is possible that our observations describe a subset of HIV virions, whereas there are other subsets that could be permeable to nucleotides. This would help explain why several reports have indicated that DNA synthesis can occur in HIV virions (25–27, 32). Another possibility is that dNTP (and rATP) concentrations are simply lower in virus particles than in cells. The local budding environment may have lower concentrations of these components, or their levels may be moderately altered in infected cells (see above), perhaps fluctuating, depending on the state of the cell. Others have reported changes in rATP levels and cellular localization during the replication of hepatitis C virus genomes (65), whereas non-uniform concentrations of rATP in cells have also been reported (66). Finally, it is possible that dNTPs packaged in the virion were lower because they were used for DNA synthesis. It has been reported that tRNA(Lys3), the primer for minus-strand DNA synthesis, is observed in three forms in the HIV virion: (i) no added dNTP, (ii) a one nucleotide dCTP extension, or (iii) a two nucleotide dCTP + dTTP extension (21). A second report found no evidence of dNTP addition to tRNA(Lys3) in the virion; hence, this possibility remains uncertain (67). Given the small number of dNTPs packaged in the virion (~1 or less based on cellular concentrations [see Introduction]), even a one or two nucleotide extension would use up most or all of the dNTPs in the virion. As the additions reported were dCTP and dTTP, this would also tend to lower those nucleotides while having no effect on others. Since all dNTPs were lowered in comparison to their cellular concentrations in our experiments (Table 1), this possibility does not explain our observations, but our results also do not directly address the possibility that tRNA(Lys3) is extended in some virions. Overall, it is possible that a combination of these factors leads to the lower observed concentration of dNTPs in virions. Note that the factors discussed above do not invoke an active mechanism to deplete or exclude nucleotides from virions, and we believe such a mechanism is highly unlikely.

A notable finding from this work was the stability of HIV virions to leakage. HIV has often been viewed as an unstable virus with relatively low infectivity. Although the latter may be true, at least with respect to temperature and freeze-thaw lability, viruses were highly stable (Fig. 4). The infectivity of HIV-1 is also relatively robust with respect to temperature as the virus slowly loses infectivity at ~50–55°C, whereas it is lost within seconds at 60°C (68, 69). This suggests that although virus infectivity is quite stable to heating, the virus is even more stable with respect to leakage, with just a trace of leakage detected at 60°C in our experiments (Fig. 4B). This finding is consistent with a previous report showing that although HIV virions lost most gp120 envelope protein during incubation at 55°C or higher, virions recovered after a 1 h incubation at 65°C retained other viral proteins, indicating that they were largely intact (70). It would be interesting to determine if virions leak dNTPs prior to losing integrity and at what temperature this occurs.

In conclusion, the presented results demonstrate that HIV virions contain dNTP levels that are at or below levels in cells. The quantities of the different dNTPs are essentially proportional to their levels in cells with dTTP >dCTP ≈ dATP >dGTP. This suggests that dNTPs are likely acquired by a passive mechanism during virus budding. The levels in virions, which may be less than 1 copy of each dNTP on average, could not support any significant DNA synthesis. The intact virion is also a stable closed environment that does not allow release and likely does not allow uptake of dNTPs, and by analogy, other small, charged molecules. Recent work showed that exposure of the HIV capsid to the cytoplasm allows dNTPs and other small molecules (e.g., Mg^2+^) to access IP6-stabilized pores on the surface of the virus capsid. This process initiates DNA synthesis that culminates with the release of the viral DNA in the nucleus (70–72). The “intact” virions observed in our work would be unable to carry out significant DNA synthesis prior to the release of the capsid in the cytoplasm. Reports of virions capable of DNA synthesis suggest that there are other virions that have properties different from those observed in this work.

## MATERIALS AND METHODS

### Materials

Deoxyribonucleotide triphosphates (dNTPs), acyclonucleotides, and T4 Polynucleotide Kinase (PNK) were from New England BioLabs. Dideoxynucleotides (ddNTPs) were from TriLink BioTechnologies. Radiolabeled ATP (γ-^32^P) was from Perkin-Elmer. G-25 spin columns were from Harvard Apparatus. All DNA oligonucleotides were from Integrated DNA Technologies (IDT). Wild-type HIV-1 reverse transcriptase (RT) (from HXB2 strain) was prepared as described (73). Aliquots of HIV-1 RT were stored frozen at −80°C, and fresh aliquots were used for each experiment. Fetal bovine serum was from R&D Systems. The p24 ELISA kit was from SinoBiologicals (Cat #: KIT11695LV3). The rATP quantification kit was from Caymen Chemical Company (Cat. # 700410). All other chemicals were from VWR, ThermoFisher Scientific, or MilliporeSigma.

### End-labeling of oligonucleotides with T4 PNK

Aptamers were 5′ end-labeled in a 50 µL volume containing 10 pmol of the aptamer, 1× T4 PNK reaction buffer (provided by the manufacturer), 10 U of T4 PNK, and 2.5 µL of (γ-^32^P) ATP (3000 Ci/mmol, 10 µCi/µL). The labeling reaction was done at 37°C for 30 min according to the manufacturer’s protocol. PNK enzyme was heat-inactivated by incubating the reaction at 75°C for 15 min. Excess radiolabeled nucleotides were then removed by centrifugation using a Sephadex G-25 column.

### Processing of virus material for dNTP assays

HEK 293T cells were grown on poly-L-lysine treated 100 mm culture dishes. Cells were rinsed with PBS and then transfected with plasmid pNL4-3 using jetOPTIMUS Polyplus transfection reagent as recommended by the manufacturer. Typically, 4–6 plates of pNL4-3-transfected and 2–3 plates of mock-transfected cells were processed. Transfections were carried out in DMEM + 10% exosome-depleted FBS (standard FBS was used for growth prior to transfection) containing 100 U/mL of penicillin and 100 µg/mL of streptomycin. Exosome depletion of FBS was carried out by centrifugation as previously described (74, 75). Forty-eight to 72 h post-transfection, media was collected and centrifuged at 1,000 × *g* for 10 min. The supernatant was filtered through a 0.45 µm 25 mm syringe (Whatman, PES) filter, and the filtrate was filtered through a second filter of the same size. The resulting filtrate was concentrated to ~3 mL using a 100 kDa MW cutoff Amicon Ultra Centrifugal Filter. The sample was placed in a 5 mL ultracentrifuge tube (5 mL, 13 × 51 mm, polypropylene) and underlaid with 1 mL of a solution containing 20 mM HEPES, pH 7.4, 150 mM NaCl, and 20% (wt/vol) sucrose. Tubes were topped off with buffer (as above minus sucrose) and centrifuged at 35,000 rpm in an SW 55Ti rotor for 90 min at 4°C. The supernatant was carefully removed until approximately 250 µL of sucrose solution remained. An additional 250 µL of sucrose minus buffer was added, and the samples were resuspended with 40 pipette strokes. Material was then concentrated using a 30 kDa cutoff 500 µL Amicon Ultra Centrifugal Filter spun until the minimum volume of retained fluid remained. The concentrated material was washed 3 times with 500 µL of sucrose minus buffer and the same centrifugation procedure. Virus and mock-transfected samples were brought to a volume of 10 µL per plate and stored at −80°C.

### Measurement of p24 levels and calculation of virion particle concentration

Virion concentrations were estimated from the amount of p24 capsid protein in the concentrated samples. A p24 ELISA (SinoBiologicals “Lentivirus [HIV-1 p24] Titer Kit” [Cat. # Cat: KIT11695LV3]) was used to measure p24 levels, and a standard conversion of 10^4^ virions per picogram of p24 was used to estimate virion concentrations.

### Aptamer assay for determining dNTP concentrations in cells and virus

Assays contained 1 nM 5´ ^32^P-labeled aptamer with the specific aptamer dependent upon which dNTP was being quantified (see Fig. 1 for a list of aptamers and the chain terminators that were used with them). Reactions were in 20 µL final volumes with the following final concentrations of components: 50 nM HIV RT (added last in 25 mM Tris-HCl, pH 8, to initiate reactions), 15 mM Tris-HCl, pH 8, 8 mM HEPES, pH 7.4, 12.5 mM KCl, 60 mM NaCl, 6 mM MgCl_2_, 0.5 mM DTT, 5 µM oligo(dT_20_), 50 nM dideoxy (ddATP or ddTTP for assays to measure dTTP or dATP, respectively) or 500 nM acyclonucleotide (acyclo ATP for assays to measure either dCTP or dGTP) (see Fig. 1 for details), 0.04% Tween 20 (omitted in some assays [see below]), specific dNTPs (0–6.3 nM as indicated), and cellular or virus material at the amounts indicated in Figures. Assays were for 25 min at 37°C which was enough time for complete extension. For assays using virus of mock-transfected control material, the material was first heated to 95°C for 2 min in 8 µL of buffer containing 20 mM HEPES, pH 7.4, 150 mM NaCl, 0.1% Tween 20. The high temperature was required to release dNTPs from the virions. We subsequently found that Tween 20 was not required and had no effect on assay results, and it was omitted in later experiments. This material was then cooled at room temperature and mixed with the components listed above to achieve the final concentrations listed. Virus and mock-transfected cellular material were used directly in the assays after processing and determining the number of virions by p24 ELISA (see above). Material for quantifying the level of dNTPs in HEK 293T cells was obtained from mock-transfected cells grown as described above, but on 100 mm plates that were not treated with poly-L-lysine. Cell media was removed, and cells were washed with PBS. Cells were trypsinized and suspended in 10 mL DMEM + 10% FBS (per plate), then pelleted by centrifugation (1,000 × *g* for 5 min). Cell pellets were resuspended in 20 mM HEPES, pH 7.4, 150 mM NaCl, centrifuged a second time, and resuspended in 200 µL (per plate) of the same buffer. A hemocytometer was used to count cells. Approximately 2 × 10^6^ cells were processed by methanol extraction as described (17). Dried samples were resuspended at 10,000 cell equivalents per µL in 20 mM HEPES, pH 7.4, 150 mM NaCl and stored at −80°C. This material was used in the dNTP assays after dilution in the resuspension buffer. All reactions were terminated by adding an equal volume of 2× gel loading buffer (8 M urea, 25 mM EDTA [pH 8], 0.025% bromophenol blue, and xylene cyanol). Samples were run on an 18% denaturing polyacrylamide gel (7 M urea, 19:1 (wt:wt) acrylamide:bis-acrylamide) (76). For dCTP and dGTP determinations, 12% formamide was added to the gels to enhance separation. Electrophoresis was stopped when the xylene cyanol dye had migrated ~two-thirds down the gel. Gels were either wrapped with saran wrap and exposed directly to a phosphorimager screen or dried to chromatography paper, and then exposed. Screens were imaged with a phosphorimager (Amersham Typhoon or Fujifilm FLA-7000) and quantitated with imager software. The concentration of particular dNTPs in virion and cell samples was determined using a standard curve constructed using dNTP concentrations typically ranging from 0.05 nM to 3.2 or 6.4 nM. The level of extended aptamer in each sample was calculated as Extended/(Total (Unextended +Extended) × 1 (corresponding to the 1 nM of total aptamer added to reactions). The minus enzyme control was used as background for Extended products. The calculated value was plotted versus the amount of dNTP added to the reaction to produce the standard curve.

### Determination of rATP concentration in virions and cells

The concentration of rATP in virions and cells was measured using an “ATP Detection Assay Kit” from Caymen Chemical Company (Cat. # 700410). The manufacturer’s instructions were followed.

### Calculation of dNTP concentrations in virions and cells

Concentration calculations were based on a HEK 293T cell volume of 1.77 × 10^−12^ L per cell and a HIV virion volume of 5.2 × 10^−19^ L per virion (12). We note that these are approximations, and there are various estimates of cell and virion volumes throughout the literature (see Introduction and Discussion). Values for dNTP and rATP from the assays described above were converted to total moles present in the 20 µL assay using the following approach: (concentration in dNTP assay [nM]) × (20 × 10^−6^ L [volume of assay]) × (1 × 10^−9^ moles/nmole) = moles of nucleotide in the assay. This value, divided by the total volume of virion or cell material (in L) added to the assay gives the concentration in M.

### Virus TCID_50_ determination

Virus TCID_50_ was determined using HeLa TZM-bl cells in a limit-dilution assay as previously described (77). TCID_50_ values for media from transfected cells and processed virions were calculated, and the TCID_50_/ng p24 was calculated as an estimate of the relative infectivity of the unprocessed vs. processed samples.

## Data Availability

Data used in preparing this article will be released upon request to the corresponding author.

## AFTER EPUB

*[This article was published on 15 July 2025 with typographical errors in the text. The errors were corrected in the current version, posted on 17 July 2025.]*

## SUPPLEMENTAL MATERIAL

Supplemental material jvi.00564-25-s0001.pdf Tables S1 and S2; Fig. S1 to S3.

ASM does not own the copyrights to Supplemental Material that may be linked to, or accessed through, an article. The authors have granted ASM a non-exclusive, world-wide license to publish the Supplemental Material files. Please contact the corresponding author directly for reuse.
