# Correction for Biswas et al., “A novel aptamer-based dNTP assay reveals that intact HIV virions are highly stable and do not contain enough dNTPs to support DNA synthesis”

**DOI:** 10.1128/jvi.02028-25

**Published:** 2026-01-07

**Authors:** Urja Biswas, Cynthia Bernal, Ruofan Wang, Jeffrey J. DeStefano

## AUTHOR CORRECTION

Volume 99, no. 8, e00564-25, 2025, https://doi.org/10.1128/jvi.00564-25. Page 13, line 18: “indicating that they were largely intact (70)” should read “indicating that they were largely intact (78).”

Page 18: The following reference was inadvertently omitted.

78. Chertova E, Bess JW, Jr, Crise BJ, Sowder IR, II, Schaden TM, Hilburn JM, Hoxie JA, Benveniste RE, Lifson JD, Henderson LE, Arthur LO. 2002. Envelope glycoprotein incorporation, not shedding of surface envelope glycoprotein (gp120/SU), is the primary determinant of SU content of purified human immunodeficiency virus type 1 and simian immunodeficiency virus. J Virol 76:5315–5325. https://doi.org/10.1128/jvi.76.11.5315-5325.2002.

