# Supplementary material for: A novel aptamer-based dNTP assay reveals that intact HIV virions are highly stable and do not contain enough dNTPs to support DNA synthesis
Source: J Virol. 2025 Jul 15;99(8):e00564-25. doi: 10.1128/jvi.00564-25 (PMC12363172; doi:10.1128/jvi.00564-25)

Supplemental Data For:

A novel aptamer-based dNTP assay reveals that intact HIV virions are highly stable and do not contain enough dNTPs to support DNA synthesis

By

Urja Biswas[1], Cynthia Bernal[1,2], Ruofan Wang[1,3], and Jeffrey J. DeStefano[1,*]

[a]Cell Biology and Molecular Genetics, Bioscience Research Building, University of Maryland, College Park, MD 20742 USA

Running Head: Low concentration of dNTPs in HIV virions

#Address correspondence to Jeffrey J. DeStefano, jdestefa@umd.edu

*Current address: REGENXBIO Inc., 9804 Medical Center Dr, Rockville, MD, 20850, USA

†Current address: Charles River Laboratories Inc., 5 Research Ct, Rockville, MD, 20850, USA

Table S1. Estimates of dNTP levels in HEK cells, lymphocytes and T cells

dATP	dGTP	dCTP	dTTP	Method	Reference
		All values are µM			
HEK 293T cells					
1.02 +/- 0.27	0.41 +/- 0.14	1.65 +/- 0.41	4.67 +/- 1.68	Radiolabeled Aptamer	This paper
2.8	2	0.8	4.5	Radiolabeled Primer	1[a]
9.7 ± 3.3	6.4 ± 1.6	5.7 ± 1.2	53.0 ± 11.2	Click Chemistry	2[b]
HEK 293 cells					
8.2 ± 0.3	10.9 ± 0.5	14.4 ± 0.2	39.4 ± 5.8	Solid Phase-Radiolabel	3[c]
Activated lymphocytes					
9.2 ± 4.5	1.52 ± 1.01	3.7 ± 2.7	16.0 ± 5.3	Mass Spec.	4,5[d]
Resting lymphocytes					
5.3 ± 2.2	0.91 ± 0.35	4.5 ± 2.9	2.9 ± 2.0	Mass Spec.	4,5[d]
Activated T cells					
3.35 ± 0.23	2.56 ± 0.36	2.89 ± 0.37	4.49 ± 0.29	Radiolabeled Primer	6[e]
Resting T cells					
1.52 ± 0.09	1.34 ± 0.09	1.68 ± 0.06	1.54 ± 0.09	Radiolabeled Primer	6[e]

a- Values were estimated from bar graph (Fig. 8D) in reference 1.
b- Values in manuscript were in "pmol/10^6 cells" and were converted to "µM" using a cell volume value of 1.77 x 10^{-12} liters/HEK 293T cell. The same value used in the current manuscript.
c- See "b" above. The cells in this case were HEK 293 not HEK 293T cells but the same estimate was used for cell volume.
d- Values from reference 5 were converted to "µM" in reference 4.
e- Values in the manuscript were in "pmol/10^6 cells" and were converted to "µM" using values of 5.95 x 10^{-13} and 2.09 x 10^{-13} liters/T cell for activated and resting (naïve) cells, respectively (7)

References

1. St Gelais C, de Silva S, Amie SM, Coleman CM, Hoy H, Hollenbaugh JA, Kim B, Wu L. 2012. SAMHD1 restricts HIV-1 infection in dendritic cells (DCs) by dNTP depletion, but its expression in DCs and primary CD4+ T-lymphocytes cannot be upregulated by interferons. Retrovirology 9:105.
2. Huang CY, Yague-Capilla M, Gonzalez-Pacanowska D, Chang ZF. 2020. Quantitation of deoxynucleoside triphosphates by click reactions. Sci Rep 10:611.
3. Landoni JC, Wang L, Suomalainen A. 2018. Quantitative solid-phase assay to measure deoxynucleoside triphosphate pools. Biol Methods Protoc 3:bpy011.
4. Gavegnano C, Kennedy EM, Kim B, Schinazi RF. 2012. The Impact of Macrophage Nucleotide Pools on HIV-1 Reverse Transcription, Viral Replication, and the Development of Novel Antiviral Agents. Mol Biol Int 2012:625983.
5. Fromentin E, Gavegnano C, Obikhod A, Schinazi RF. 2010. Simultaneous quantification of intracellular natural and antiretroviral nucleosides and nucleotides by liquid chromatography-tandem mass spectrometry. Anal Chem 82:1982-9.
6. Diamond TL, Roshal M, Jamburuthugoda VK, Reynolds HM, Merriam AR, Lee KY, Balakrishnan M, Bambara RA, Planelles V, Dewhurst S, Kim B. 2004. Macrophage tropism of HIV-1 depends on efficient cellular dNTP utilization by reverse transcriptase. J Biol Chem 279:51545-53.
7. Waugh RE, Lomakina E, Amitrano A, Kim M. 2023. Activation effects on the physical characteristics of T lymphocytes. Front Bioeng Biotechnol 11:1175570.

Table S2: Infectivity of HIV-1 processed virions vs. HIV-1 directly from culture media

[a]*Experiment #- sample type*	[b]*$TCID_{50}$/ml*	[c]*p24 (ng/ml)*	[d]*($TCID_{50}$/ml)/ (ng/ml p24)*
1- cell media	557	760	0.73 (3-fold)
1- virion	7.90×10^3	33000	0.24
2- cell media	3.16×10^3	430	7.3 (2.5-fold)
2- virion	1.16×10^5	40000	2.9
3- cell media	3.16×10^3	800	4 (2.2-fold)
3- virion	7.91×10^4	43000	1.8

a- Three independent experiments. Cell media was taken directly from transfected cells, centrifuged at 1000 x g for 10 min and supernatant used directly for assays. Virions were purified and concentrated as described in the main text.
b- Calculated using HeLa TZM-bl cells in a limit dilution assay as described in the main text.
c- Quantified using p24 ELISA (SinoBiologicals "Lentivirus (HIV-1 p24) Titer Kit" (Cat. # Cat: KIT11695LV3).
d- Values are (column 2)/(column 3). Parentheses: fold greater infectivity of cell media material.

Fig. S1. Aptamer assay conducted with fluorescein labeled aptamer. The 38-nt aptamer used to quantify dTTP (38NT-2,4-methyl-dTTP) was used to measure standard concentrations of dTTP (as indicated). Aptamer with a fluorescein group attached at the 5' end was used instead of radiolabeled aptamer. The assay was conducted as described in the main text with the following changes: aptamer concentration was 40 nM, HIV RT was 100 nM, and ddATP was 150 nM. All assays were visualized using an Amersham Typhoon platform that supports phosphor imaging and fluorescence detection.

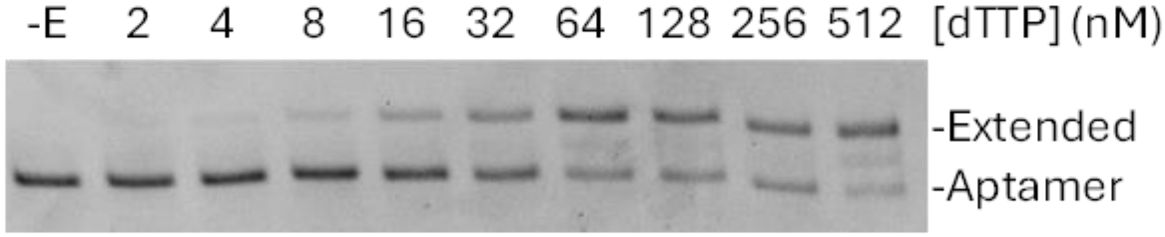

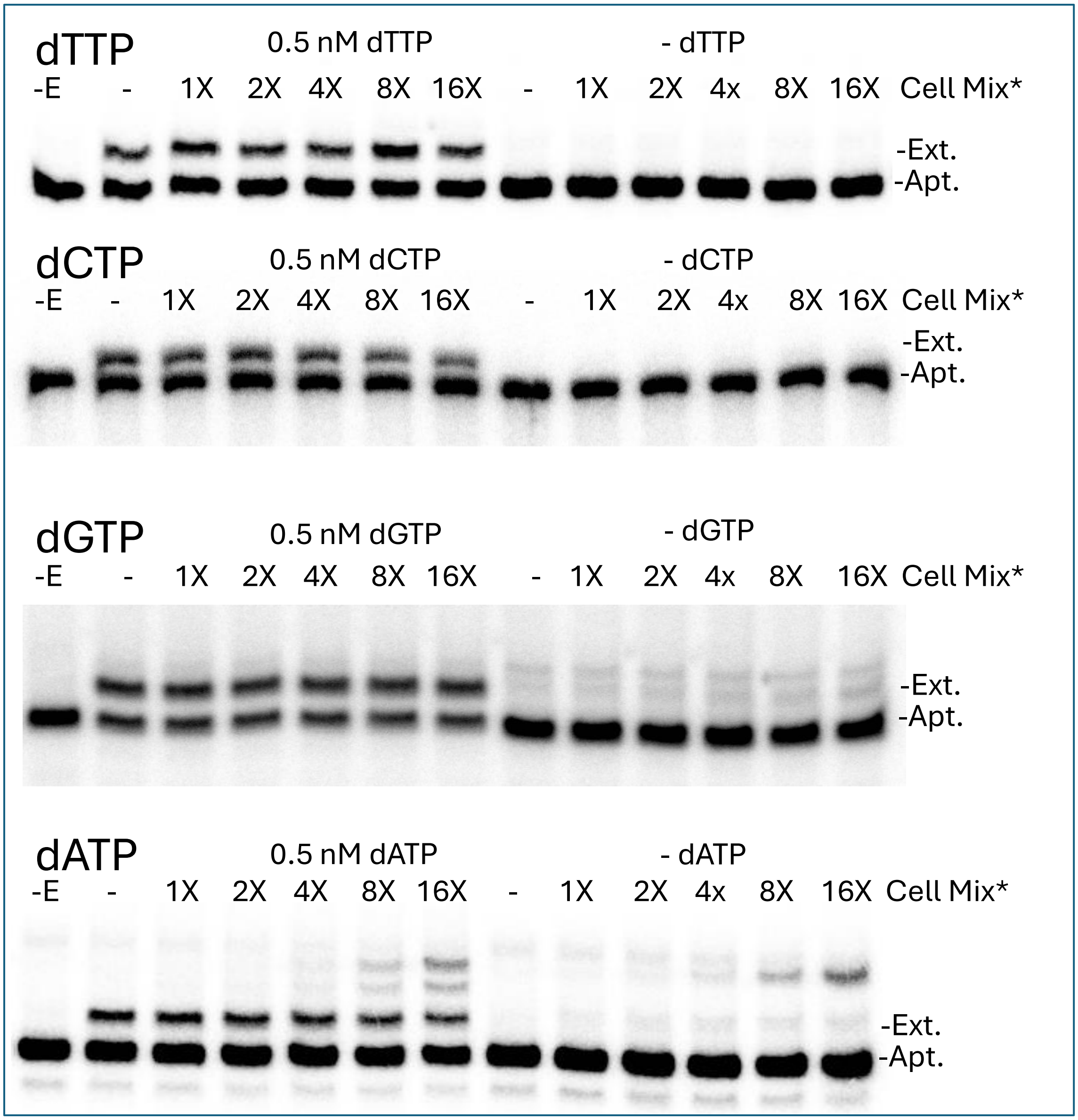

Fig. S2. Aptamer dNTP assays conducted in the presence of competing cellular nucleotides. The 38-nt aptamer (38NT-2,4-methyl-dNTP) used to quantify the indicated dNTP was used in each assay at the standard 1 nM concentration. Assays were performed as described in the main text except that a mixture containing competing cellular rNTPs and dNTPs was added at various concentrations (i.e., 1X, 2X, etc.) as indicated. Assays were performed using 0.5 nM of the dNTP being measured and this dNTP was excluded from the cellular nucleotide mix. A second set of assays (right side of panels) was conducted with the same mix but in the absence of the 0.5 nM added dNTP to access aptamer extension in the presence of the cellular mix and absence of the correct dNTP. Cellular mixes were roughly based on the results shown in Table 1 of the main text. This was used to set dNTP concentrations in the cell to 1.02, 0.41, 1.65, and 4.67 µM for dATP, dGTP, dCTP, and dTTP, respectively. rATP was set at 1 mM (1000 µM) and the other 3 rNTPs, which were not measured in our assays but are known to be at lower concentrations than rATP in cells, were set at ½ that value (0.5 mM). Using a 0.5 nM dTTP assay as an example, relative concentrations of other nucleotides would be: 0.11 (0.5/4670 x 1020), 0.04, and 0.18 nM for dATP, dGTP, and dCTP, respectively. For rATP, the value would be 0.11 µM (0.5/4670 x 1000) and the other rNTPs would be 0.06 µM. This was set to a 1X cellular concentration for the dTTP aptamer assay and these concentrations were doubled up to 16X in reactions as indicated. For assays of other nucleotides, the cellular nucleotide mix omitted the measured nucleotide and calculations for other nucleotides were based on what they would have been if the measured dNTP was at 0.5 nM. Lanes labeled "-" contained no added cell mix and "-E" lanes contained no reverse transcriptase enzyme.

Figure S3. Aptamer dNTP assay shows Control samples contain very low but detectable dTTP. The 38-nt aptamer used to quantify dTTP (38NT-2,4-methyl-dTTP) was used to measure dTTP in Hek 293T cell control samples using a slightly modified version of the assay described in the main text. Controls were Hek 293T cells that were mock transfected in the absence of pNL4-3 viral plasmid, then processed along with the virus transfected samples. Controls were matched to virus transfected samples on a per plate basis with one 100 mm plate of control sample equivalent to one 100 mm plate of virus transfected sample. The assays here contained 10 µl of Control cell sample while typical assays for measurement of virion dNTPs contained 1-8 µl (see main text). Reactions conditions were modified slightly to accommodate the larger volume of material. A standard assay with different amounts of dTTP (lanes on left) was also conducted under these conditions. The conditions were: 20 µL final volume with the following final concentrations of components: 50 nM HIV RT (added last in 25 mM Tris-HCl, pH 8, to initiate reactions), 15 mM Tris-HCl, pH 8, 10 mM HEPES, pH 7.4, 75 mM NaCl, 6 mM $MgCl_2$, 0.5 mM DTT, 5 µM oligo(dT_{20}), 50 nM dideoxy ATP, and 0.04% Tween 20. Assays were for 25 min at 37°C and were processed as described in the main text. *Refers to controls from 4 independent experiments. C^1, C^3 and C^4 showed detectable levels of dTTP, although they did not rise to the level of the lowest standard. No dTTP was detected in C^2. Lanes with no dNTP added are shown in the absence (-E) and presence (+E) of the HIV RT enzyme used to extend the aptamer.

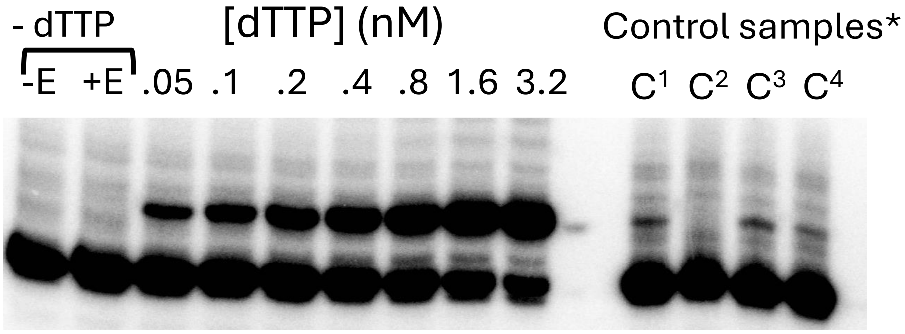

Supplement: Supplemental material — Tables S1 and S2; Fig. S1 to S3. [file jvi.00564-25-s0001.pdf]
